# Lopinavir/r no longer recommended as a first-line regimen: a comparative effectiveness analysis

**DOI:** 10.7448/IAS.17.1.19070

**Published:** 2014-09-25

**Authors:** Valérie Potard, David Rey, Isabelle Poizot-Martin, Saadia Mokhtari, Christian Pradier, Willy Rozenbaum, Françoise Brun-Vezinet, Dominique Costagliola, on behalf of the FHDH ANRS CO4 (French Hospital Database on HIV, Agence Nationale de Recherches sur le Sida, cohort 4)

**Affiliations:** 1 Sorbonne Universités, UPMC Univ Paris 06, UMR_S 1136, Institut Pierre Louis d'Epidémiologie et de Santé Publique, Paris, France; 2 INSERM, UMR_S 1136, Institut Pierre Louis d'Epidémiologie et de Santé Publique, Paris, France; 3 INSERM Transfert, Paris, France; 4 Hôpitaux Universitaires de Strasbourg, Strasbourg, France; 5 Aix Marseille Univ, APHM Sainte-Marguerite, Service d'Immuno-hématologie clinique, INSERM U912 (SESSTIM) Marseille, Marseille, France; 6 Service de maladies infectieuses et tropicales Hôpital Nord AP-HM, Marseille, Marseille, France; 7 Département de Santé Publique, CHU Nice, Nice, France; 8 St-Louis CHU Paris, AP-HP, UPMC Univ Paris 06, Paris, France; 9 Laboratoire de Virologie, Assistance Publique-Hôpitaux de Paris (AP-HP), Groupe Hospitalier Bichat-Claude Bernard, HUPNVS, Université Paris Diderot, Paris, France

**Keywords:** first-line therapy, HIV infection, antiretroviral therapy, effectiveness, propensity score

## Abstract

**Introduction:**

We compared the effectiveness of tenofovir/emtricitabine (TDF/FTC) combined with either lopinavir/r (LPV/r) or another recommended third drug in the 2010 French guidelines in antiretroviral-naïve patients starting combination antiretroviral therapy in 2004–2008 in the French Hospital Database on HIV.

**Methods:**

The outcomes were stop or switch of the third component, viral load (VL) <500 copies/ml, an increase of at least 100 CD4 cells/mm^3^, AIDS-defining event and non-AIDS-defining hospitalization or death. Propensity scores were estimated by logistic regression based on the clinical centre and other confounders. In each clinical centre, each patient initiating LPV/r was matched with a patient initiating another third drug (efavirenz or atazanavir/r) and having a close propensity score. Cox's proportional hazards models were then used, with treatment as covariate. Time was right-censored at four years.

**Results:**

1269 patients started LPV/r plus TDF/FTC, and 890 could be matched to 890 patients receiving another third drug. Baseline characteristics were well balanced between these two groups. LPV/r was associated with a higher risk of third drug stop (hazard ratio (HR): 1.69; 95% confidence interval (CI), 1.42–2.00) and with less rapid viral suppression (HR: 0.83; 95% CI, 0.72–0.95). There was no difference in the time required for a CD4 cell increment of at least 100/mm^3^, or to the occurrence of an AIDS-defining event. Non-AIDS-defining hospitalizations or deaths were more frequent with LPV/r (HR: 1.79; 95% CI, 1.33–2.39).

**Conclusions:**

For first-line therapy, in this observational setting, TDF/FTC plus LPV/r were less durable than TDF/FTC plus another recommended third drug, led to a less rapid viral suppression and were associated with a higher risk of non-AIDS morbidity.

## Introduction

The primary aims of antiretroviral therapy (ART) for HIV infection are to reduce morbidity and to prolong life by reducing viral load (VL) and increasing the CD4+ T-cell count with minimal toxicity/adverse event (AE). Combination antiretroviral therapy (cART) comprising two nucleoside reverse transcriptase inhibitors (NRTI) and a third drug, either a ritonavir-boosted protease inhibitor (PI) or a non-nucleoside reverse transcriptase inhibitor (NNRTI), has shown good virologic and immunologic efficacy in many randomized clinical trials [1–9].

The efficacy of ritonavir-boosted PI-containing regimens relies on the additional dose of ritonavir used as a pharmacokinetic booster. Lopinavir/r (LPV/r) has been the boosted PI of choice for many years, and was the only such co-formulation available in 2011. Other boosted PI-based regimens with a more favourable toxicity profile became available as early as 2004.

Between 2004 and 2008, several randomized trials compared various antiretroviral (ARV) drugs in treatment-naïve patients [3–9]. In 2008, based on the results of these trials, American and European guidelines recommended efavirenz (EFV), LPV/r, atazanavir/r (ATV/r) or fosamprenavir/r (FPV/r)-based regimens as preferred options for ART-naïve patients [10,11]. In 2012, EFV and ATV/r were still recommended in American and European guidelines, but FPV/r was listed as an alternative. LPV/r was still recommended for ART-naïve patients in 2010 European guidelines but was listed as an alternative in US guidelines. These recommendations were based on virologic efficacy and, to a lesser extent, on the CD4 cell increment and tolerability.

Since ARV drugs are generally not assessed on clinical criteria, because of limited-duration randomized controlled trials and low event frequency, it is important to know whether the various recommended ARV drugs have the same impact on the basis of clinical criteria defined as AIDS-related morbidity or severe non-AIDS morbidity such as malignant, cardiovascular and infectious diseases. In addition, because patients enrolled in clinical trials are selected, and for example individuals with severe comorbidities are often excluded, clinical trial findings can be less generalizable than observational study findings, so it is important to assess the effectiveness of different treatments in routine care settings, complementarily with clinical trials [12].

The purpose of this study was to assess whether LPV/r was still a valid option for ART-naïve patients. Using a comparative effectiveness approach, LPV/r was compared to other recommended third drugs (EFV and ATV/r) in the 2010 French guidelines, always in combination with tenofovir/emtricitabine (TDF/FTC), using five criteria for effectiveness: the durability of the third component, virologic and immunologic responses, AIDS morbidity and severe non-AIDS morbidity.

## Methods

### Patients and data sources

The FHDH-ANRS CO4 (French Hospital Database on HIV, Agence Nationale de Recherches sur le Sida, cohort 4) is an open hospital cohort study created in 1992. It includes 70 hospitals belonging to 26 Regional HIV/AIDS Coordination Centres (COREVIHs) located in mainland France and French overseas territories. Patients are eligible if they have documented HIV-1 or HIV-2 infection and give their written informed consent to participate. Data are collected prospectively by trained research assistants on standardized forms which include the transmission group, biological markers such as the CD4 cell count and plasma HIV RNA level, clinical manifestations, the nature and starting date of prescribed treatments, and death. The reasons for ARV drugs stopping have been collected since 2005.

### Study population

This study was restricted to ARV-naïve adults and adolescents living with HIV and with a VL >500 copies/ml, who started a first-line three-drug cART regimen consisting of TDF/FTC in every case, plus LPV/r, ATV/r or EFV. The study period for cART initiation was 2004–2008. We selected 2004 as this was the year atazanavir became available, and 2008 in order to provide four-year results. Women with reported pregnancy and patients infected less than six months previously were not eligible, as they qualified for specific therapeutic strategies.

### Statistical analysis

The baseline for all analyses was the date of treatment initiation. The following baseline characteristics were described according to the prescribed regimen: clinical centre, age, sex transmission, sub-Saharan origin, hepatitis C virus co-infection, time after diagnosis of HIV-1 infection (<1 year or ≥1 year), AIDS status, year of treatment initiation, baseline CD4 cell count (log_2_) and VL (log_10_) and cotrimoxazole prophylaxis.

Rather than comparisons of LPV/r with each other recommended third drug, we chose to compare the LPV/r regimen to the other two regimens combined, after checking that ATV/r and EFV had similar effectiveness in our study. Combining ATV/r and EFV into one group also allows one to diminish the selection bias, the impact of unmeasured confounders in an observational setting, and to increase the statistical power in the comparison. In addition, as the backbone may play a role in the outcomes of interest, we only studied patients initiating with TDF/FTC.

Biological and clinical responses were compared by using the propensity score method, which is used in observational studies to control for systematic differences between nonrandomized treatment groups, thereby reducing the indication bias and confounding factors [13–15]. The propensity score for each subject, defined as the conditional probability of receiving LPV/r given his or her individual covariates, was estimated from a logistic regression model that included baseline characteristics and the clinical centre. To take into account a possible clinical centre effect, each patient initiating LPV/r was matched with a patient who started on another third drug (EFV or ATV/r) in the same clinical centre and who had a close propensity score (within ±0.04). Absolute standardized differences were used to compare the balance in baseline characteristics between two treatment groups [16].

We evaluated four-year Kaplan-Meier estimates for all of the following outcomes: third component stop or switch; virologic success, defined as ever achieving a VL <500 copies/ml while still receiving the drug; a gain of at least 100 CD4 cells/mm^3^; AIDS morbidity (AIDS-defining event or death from an AIDS-defining event); and severe non-AIDS morbidity (non-AIDS hospitalization or death from a non-AIDS cause). The choice of threshold (500 copies/ml) was due to assay technique changes over time in the study period. Cox's proportional hazards models were used, including the type of treatment as the only variable in the model, in the group of patients initiating LPV/r and their matched counterparts initiating another third drug, in order to compare outcomes between the two matched groups. The other third drug (EFV or ATV/r) served as the reference. Time was right-censored at four years. As a change in the third drug could be considered as a competing event (such patients are likely to be those experiencing a slower reduction in VL or a slower increase in the CD4 cell count), a competing-risk approach was adopted for virologic and immunologic responses. In this approach, when the third drug was changed, follow-up was right-censored at the date of the patient's last visit during the four-year follow-up period. This approach ensures that no endpoint can be recorded during the period between the treatment change and the end of the four-year follow-up period, thus avoiding a situation in which the majority of patients change their third drug and achieve a VL reduction (or a gain in CD4 cells) while on this alternative treatment. For both clinical endpoints, we used an intention-to-continue-treatment approach, ignoring treatment changes. We also described the reasons for stopping or switching the third drug as follows: ineffective treatment, intolerance and/or toxicity/adverse event, treatment simplification and other reasons. We used SAS software (v9.2; SAS Institute Inc., Cary, NC, USA) for all statistical analyses.

## Results

### Patients enrolled and baseline characteristics

**Figure 1:**
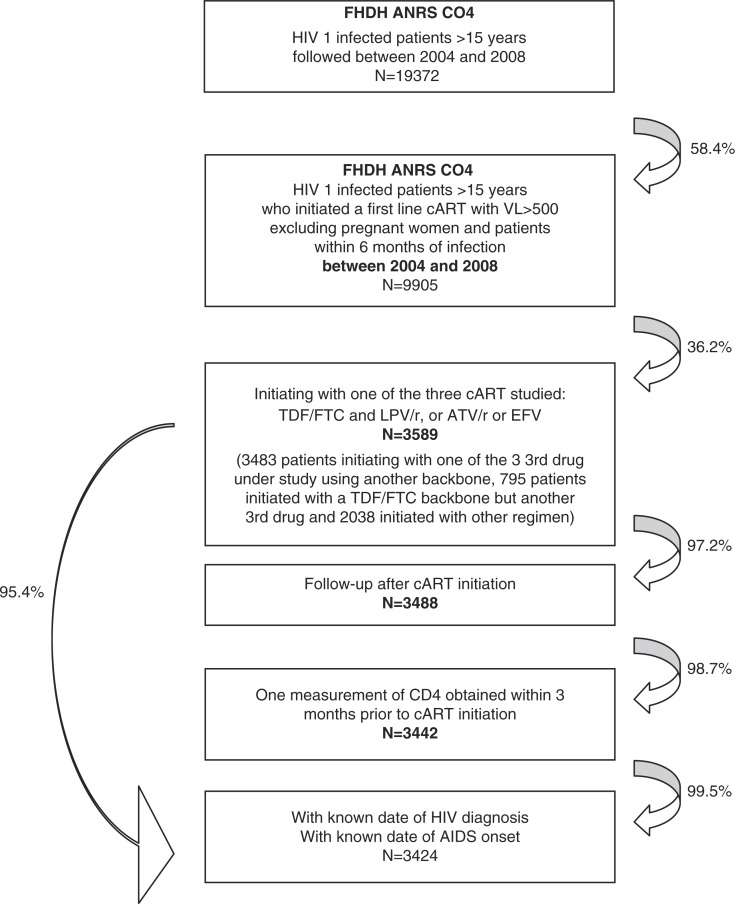
Patient flow.

The flow chart of the study is shown in Figure 1. A total of 3424 patients were included, of whom 1269 started LPV/r plus TDF/FTC and 2155 started EFV (*n*=1441) or ATV/r (*n*=714) plus TDF/FTC. Several characteristics of the two initial treatment groups were different (Table 1, Unmatched). The propensity scores of the two treatment groups showed substantial overlap Supplementary Figure 1), allowing 890 patients (70%) to be matched. Unmatched patients (*n*=379) had more advanced HIV infection, with lower CD4 cell counts (median 81 vs. 223), higher VL (median 5.2 vs. 4.9) and more frequent prior AIDS-defining event (40% vs. 17%). Absolute standardized differences comparing baseline characteristics between the two groups in the unmatched and matched samples are reported in Supplementary Figure 2. After propensity score matching, the standardized differences of all the characteristics were less than 10%, indicating a successful balance.

**Table 1:** Baseline characteristics of patients

	Unmatched patients			Matched patients	
	
	LPV/r (*n*=1269)	EFV, ATV/r (*n*=2155)	*p*	LPV/r (*n*=890)	EFV, ATV/r (*n*=890)
Age (years) (median [IQR])	40.0 [33.5–46.8]	39.5 [32.9–46.7]	0.41	39.6 [33.5–46.5]	39.8 [32.6–47.0]
Sex transmission			**0.0004**		
MSM	418 (33%)	860 (40%)		304 (34%)	324 (36%)
IDU	75 (6%)	94 (4%)		52 (6%)	50 (6%)
Heterosexual men	329 (26%)	564 (26%)		233 (26%)	221 (25%)
Heterosexual women	340 (27%)	490 (23%)		229 (26%)	223 (25%)
Other men	71 (6%)	94 (4%)		46 (5%)	48 (5%)
Other women	36 (3%)	53 (3%)		26 (3%)	24 (3%)
Sub-Saharan origin	317 (25%)	426 (20%)	**0.0004**	228 (26%)	217 (24%)
HCV positive	119 (9%)	186 (9%)	0.46	90 (10%)	77 (9%)
Time since HIV diagnosis <1 year	710 (56%)	859 (40%)	**<0.0001**	428 (48%)	425 (48%)
CD4 cell count (/mm^3^) (median [IQR])	191 [71–280]	260 [180–326]	**<0.0001**	223 [122–300]	237 [146–303]
Plasma HIV-1 RNA (log_10_ copies/ml) (median [IQR])	5.00 [4.44–5.50]	4.78 [4.28–5.20]	**<0.0001**	4.89 [4.33–5.40]	4.88 [4.35–5.28]
Prior AIDS	305 (24%)	285 (13%)	**<0.0001**	155 (17%)	157 (18%)
Prophylaxis (yes)	565 (45%)	605 (28%)	**<0.0001**	336 (38%)	338 (38%)
Year of starting cART			**<0.0001**		
2004	16 (1%)	81 (4%)		13 (2%)	10 (1%)
**2**005	77 (6%)	247 (12%)		61 (7%)	65 (7%)
2006	329 (26%)	515 (24%)		225 (25%)	223 (25%)
**2**007	417 (33%)	497 (23%)		264 (30%)	261 (29%)
2008	430 (34%)	815 (38%)		327 (37%)	331 (37%)

LPV/r=lopinavir/r; EFV=efavirenz; ATV/r=atazanavir/r; IQR=interquartile range; MSM=men who have sex with men; IDU=injectable drug users; HCV=hepatitis C virus; cART=combination antiretroviral therapy; Bold values indicate that P is significant (<0.05).

The third drug was EFV in 614 cases (69%) and ATV/r in 276 cases (31%). Median follow-up was 42.0 months (interquartile range: 26.4–55.2).

### Outcomes

No difference was found between EFV and ATV/r on any of the outcomes. Figure 3 shows Kaplan-Meier plots of the different outcomes and corresponding hazard ratios (HRs) for LPV/r compared to another third drug up to four years after treatment initiation.

### Durability of treatment

**Figure 2:**
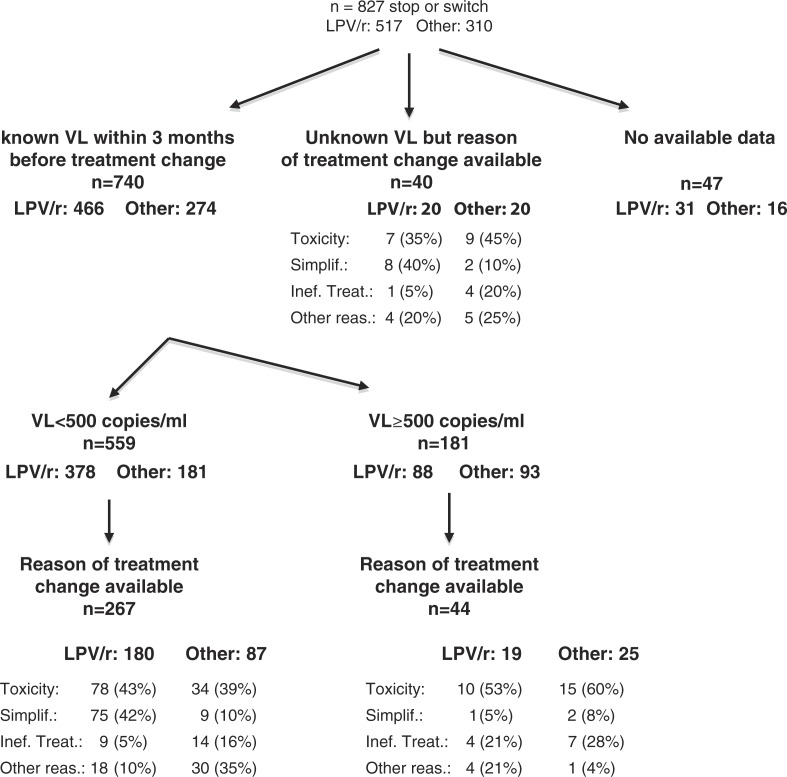
Stop or switch treatment flow.

Of the 890 matched patients initiating LPV/r, 517 patients changed their treatment, and of the 890 patients initiating another third drug, 310 patients changed their treatment. The flow chart in Figure 2 shows the number of documented treatment stops according to information that has been collected (known VL within three months before the stop and the reason for stop available in the database).

Among the 267 patients who changed their third drug after VL <500 copies/ml and for whom the reason for the change was available, these reasons differed between the treatment groups (*p*<0.0001). Among toxicity/adverse event the proportion of patients with lipid disorders who switched after initiating LPV/r was 8% versus 0% for patients initiating another third drug (*p*=0.007). The proportion of switch for simplification was greater for patients initiating LPV/r than for patients initiating another third drug. Among the 44 patients who changed their third drug with VL ≥500 copies/ml, the reasons for the change did not differ between the treatment groups (*p*=0.36).

**Figure 3:**
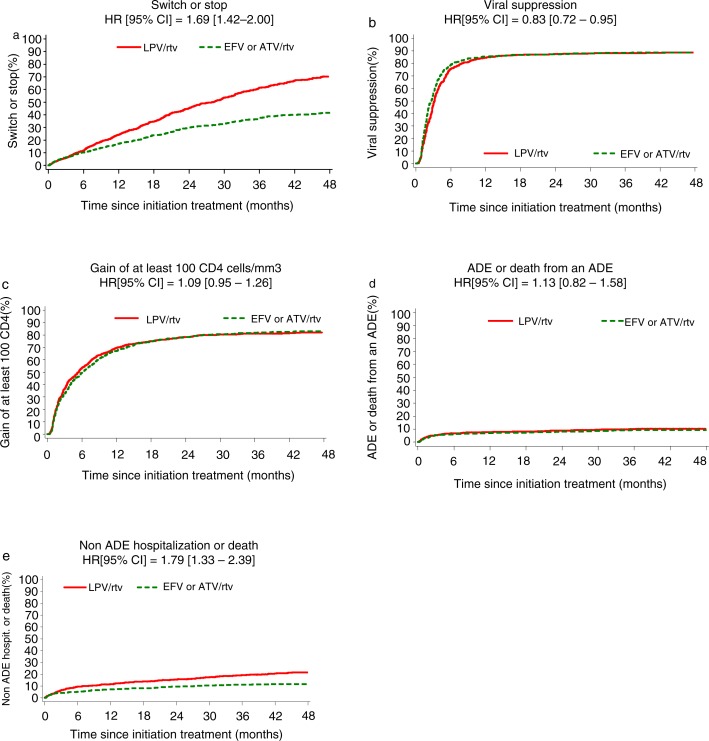
Kaplan-Meier plots showing the time (a) to stop or switch the third component, (b) to achieve a virologic response of viral load <500 copies/ml, (c) to gain at least 100 CD4 cells/mm^3^, (d) to the occurrence of an adverse event (AE) or death from an AE, and (e) to the occurrence of a non-AE hospitalization or death.

The estimated proportion of patients who had changed at month 48 was 70% with LPV/r versus 42% with the other third drugs. The LPV/r-containing regimen was significantly less durable than the other regimens (HR=1.69; 95% confidence interval (CI), 1.42–2.00) (Figure 3a). The estimated proportion of patients who changed their treatment after achieving virologic success was 63% with LPV/r versus 29% with the other third drugs (HR=2.10; 95% CI, 1.67–2.65).

### Virologic outcome

VL fell below 500 copies/ml in 759 patients on LPV/r and in 765 patients on the other third drugs. LPV/r was associated with less rapid viral suppression (*p*<0.0001) (Figure 3b). At month three, the rate of virologic success was 48% with LPV/r versus 56% with the other third drugs (*p*<0.0001); at month six, this rate was 76% with LPV/r versus 79% with the other third drugs (*p*=0.06); whereas the difference was no longer significant at later times. Overall, the proportion of virologic success (ever achieving a VL <500 copies/ml while still receiving the drug) was lower for LPV/r than for another third drug (HR=0.83; 95% CI, 0.72–0.95).

### Immunologic outcome

The CD4 cell count rose by at least 100/mm^3^ in 658 patients initiating LPV/r and in 663 patients initiating another third drug. There was no difference between LPV/r and the other third drugs with respect to the time required for the CD4 cell count to rise by at least 100/mm^3^ (83% for the two groups at 48 months) (Figure 3c).

### Clinical outcomes

During the 48 months of follow-up, 161 patients experienced at least one AIDS-defining event (85 in the LPV/r group and 76 in the other group). Overall, 121 patients experienced one AIDS-defining event, 31 patients experienced two, seven patients experienced three and two patients experienced four. Among the 212 AIDS-defining event, the five most frequent were tuberculosis (*n*=43 individuals and 52 events; 18 patients with extrapulmonary tuberculosis, 16 with pulmonary tuberculosis and 9 with both extrapulmonary and pulmonary tuberculosis during the follow-up), Kaposi's sarcoma (*n*=33), *Pneumocystis jiroveci pneumonia* (*n*=23), cerebral toxoplasmosis (*n*=16) and oesophageal candidiasis (*n*=15). There was no difference in the occurrence of AIDS-defining event or deaths from an AIDS-defining event between LPV/r and the other third drugs (10% vs. 9% at 48 months) (Figure 3d).

During the 48 months of follow-up, 237 patients experienced at least one non-AIDS-hospitalization or death (153 with LPV/r and 84 with the other third drugs). One-third of hospitalizations were for non-AIDS-defining infection (*n*=91). The other four most common reasons for non-AIDS hospitalization were chronic viral hepatitis (*n*=13), non-AIDS-efining malignancies (*n*=12), haematologic disorders (*n*=12) and psychiatric disorders (*n*=9). Four patients on LPV/r and two of the patients on another third drug were hospitalized for cardiovascular disease. LPV/r was associated with a higher risk of non-AIDS morbidity with an HR of 1.79 (95% CI, 1.33–2.39) compared to the other third drugs (21% vs. 12% at 48 months) (Figure 3e).

## Discussion

In this large observational study, after taking into account the propensity for receiving LPV/r, LPV/r was associated with shorter treatment durability, less rapid viral suppression and a higher risk of severe non-AIDS morbidity when compared to other recommended third drugs. No difference was found between LPV/r and the other third drugs with respect to immunologic efficacy or AIDS morbidity.

Two strengths of our study were the large sample size and the use of propensity scores to control for confounding factors in this observational setting. One limitation is that 30% of patients receiving LPV/r could not be matched. However, their clinical outcomes tended to be worse, and this cannot therefore explain the worse clinical outcomes we observed among the LPV/r-treated patients in our analysis.

Although the propensity score method cannot control for unmeasured confounders, the most important ones in terms of prognosis were taken into account in our analyses, except for adherence as no adherence data are available in the FHDH. Our decision to compare LPV/r to all other recommended third drugs combined instead of separately limited the possibility of a strong unmeasured confounder. Our choice to combine EFV and ATV/r was clinically justified because these drugs are recommended “third drugs” in the guidelines. In the ACTG A5202 randomized trial, ATV/r was as efficacious and well tolerated as EFV [6].

We restricted our study to a single NRTI backbone (TDF plus FTC) in order to be able to compare the impact of the third drug. Indeed, it has been reported that the choice of NRTI backbone is a significant predictor of virologic success and treatment failure [17–19]. Finally, in our analysis of biological responses, any change in the third component was considered to represent treatment failure. This approach may better reflect the effectiveness of the third component, as changes in treatment may be due to either inefficiency or intolerance. Unfortunately, reason for treatment change was missing in 57.6% of the 827 stops or switches of the third component.

The rate of stop or switch was larger in patients receiving LPV/r than in patients receiving another third drug, in keeping with the results of the CORIS study [20]. Here, the most frequent reason for treatment changes was toxicity/adverse event, as in other observational studies [20–22]. Toxicity remains a major cause of treatment discontinuation, and for LPV/r the simplification is also an important reason to stop this drug. Many patients who changed their treatment after achieving virologic success did so for reasons of simplification (42% in the LPV group). This reflects a strategy in which a PI/r-based regimen is prescribed first to ensure less resistance in case of failure and is then replaced by a simpler regimen that is also more tolerable in the long term [23]. The great increase in the number of switching events in patients on LPV/r over time could indicate the availability of novel and simpler therapies gaining popularity among practitioners. The rate of treatment discontinuation tended to be higher in this observational study than in clinical trials, possibly because patients with severe comorbidities and biological abnormalities are often excluded from clinical trials [2,4,24].

The initial difference in virologic efficacy before six months, between LPV/r and the other third drugs, disappeared after 12 months. A comparative effectiveness study also showed that differences between regimens in terms of virologic efficacy were not as pronounced over time [25]. Likewise, in the NORTHIV trial, EFV was superior to LPV/r at 48 weeks (69% vs. 86%), but at 144 weeks there was no significant difference between the two groups [26]. However, in the CASTLE randomized trial, the proportions of patients who achieved a VL <50 copies/ml were similar in the LPV/r and ATV/r groups at week 48 (76% vs. 78%), but fewer patients receiving LPV/r had HIV RNA <50 copies/ml at week 96 compared to patients receiving ATV/r (68% vs. 74%) [3]. In the ACTG 5142 randomized trial, a greater early effect of EFV compared with LPV/r on suppression of HIV RNA levels (<50 copies/ml) has been reported at week 96 (77% vs. 89%) [5]. The lack of difference at two years in our study may have been due to the use of a less strict virologic endpoint (HIV RNA <500 copies/ml) than in recent clinical trials.

Despite the more rapid virologic efficacy of the other third drugs compared to LPV/r during the first six months of our study, the gain in CD4 cells was similar. In clinical trials, no significant difference in the CD4 cell gain from baseline was found at week 48 between the ATV/r and LPV/r and the EFV and LPV/r groups [3,5,9,26].

Regarding clinical outcomes, although there was no significant difference in AIDS morbidity between the LPV/r and comparator regimens, we found that LPV/r was associated with a higher risk of non-AIDS morbidity. Clinical trials lacked sufficient power to detect differences in clinical outcomes [2,4,9,24,26]. Causes of hospitalization were similarly distributed in the two groups, although their frequency was higher with LPV/r. Although LPV/r has been linked to lipid disorders, we noted no increase in the risk of hospitalization for cardiovascular diseases among patients receiving LPV/r [2,4,9,24]. Among the causes of hospitalization, we noted a lot of non-AIDS-defining infection. Higher risk of non-AIDS morbidity among patients receiving LPV/r could be perhaps explained by the slower rate of reduction in VL that we observed after initiating treatment, which could affect non-AIDS-defining infection occurrence in these patients.

## Conclusions

This study shows that first-line cART with LPV/r is less durable, provides less rapid viral suppression and is associated with more frequent severe non-AIDS clinical events than other recommended third drugs. These findings support recommendations that LPV/r should be considered as an alternative rather than as a preferred drug for cART-naïve patients, as done in the 2013 French and European guidelines where LPV/r was listed as an alternative [11,27].

## Supplementary Material

Lopinavir/r no longer recommended as a first-line regimen: a comparative effectiveness analysis
