# Supplementary material for: Lopinavir/r no longer recommended as a first-line regimen: a comparative effectiveness analysis
Source: J Int AIDS Soc. 2014 Sep 25;17(1):19070. doi: 10.7448/IAS.17.1.19070 (PMC4176690; doi:10.7448/IAS.17.1.19070)
Supplement: Lopinavir/r no longer recommended as a first-line regimen: a comparative effectiveness analysis [file JIAS-17-19070-s001.pdf]

Supplementary material

Figure S1. Distribution of propensities of score according to treatment

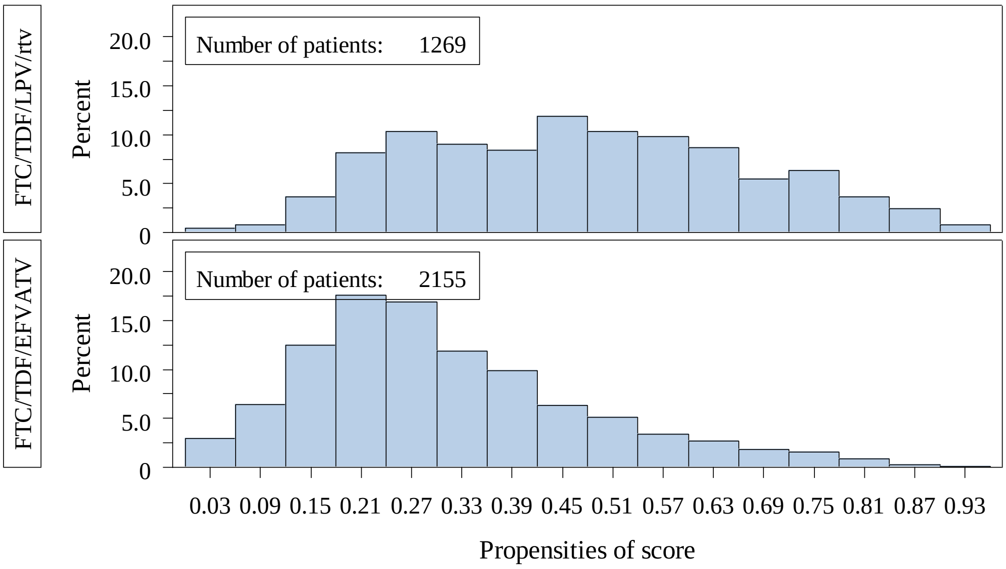

Table S1. Unmatched and matched patients in each Corevih

	Unmatched patients			Matched patients	
	LPV/r (n=1269)	EFV,ATV/r (n=2155)	P	LPV/r (n=890)	EFV,ATV/r (n=890)
**Corevih**			**<0.0001**		
IDF Centre	128 (10%)	276 (13%)		112 (13%)	112 (13%)
IDF Est	193 (15%)	352 (16%)		177 (20%)	177 (20%)
IDF Nord	76 (6%)	128 (6%)		49 (6%)	49 (6%)
IDF Ouest	106 (8%)	235 (11%)		88 (10%)	88 (10%)
IDF Sud	152 (12%)	283 (13%)		112 (13%)	112 (13%)
ALSACE	44 (4%)	36 (2%)		28 (3%)	28 (3%)
Arc Alpin	25 (2%)	17 (1%)		10 (1%)	10 (1%)
Auvergne Loire	19 (2%)	32 (2%)		9 (1%)	9 (1%)
Basse Normandie	22 (2%)	33 (2%)		12 (1%)	12 (1%)
Bourgogne	4 (0%)	43 (2%)		4 (0%)	4 (0%)
Bretagne	21 (2%)	35 (2%)		16 (2%)	16 (2%)
Centre	15 (1%)	17 (1%)		7 (1%)	7 (1%)
Franche Comté	8 (1%)	13 (1%)		3 (0%)	3 (0%)
Haute Normandie	22 (2%)	24 (1%)		9 (1%)	9 (1%)
Languedoc Roussillon	38 (3%)	21 (1%)		14 (2%)	14 (2%)
Lorraine	32 (3%)	43 (2%)		20 (2%)	20 (2%)
Midi-Pyrénées	16 (1%)	56 (3%)		14 (2%)	14 (2%)
Nord Pas de Calais	2 (0%)	5 (0%)		1 (0%)	1 (0%)
PACA Est	41 (3%)	36 (2%)		22 (3%)	22 (3%)
PACA Ouest	64 (5%)	160 (7%)		59 (7%)	59 (7%)
Pays de la Loire	55 (4%)	37 (2%)		26 (3%)	26 (3%)
Vallée du Rhône	109 (9%)	65 (3%)		54 (6%)	54 (6%)
GUADELOUPE	40 (3%)	84 (4%)		28 (3%)	28 (3%)
GUYANNE	7 (1%)	72 (3%)		7 (1%)	7 (1%)
MARTINIQUE	12 (1%)	19 (1%)		2 (0%)	2 (0%)
REUNION	18 (1%)	33 (2%)		7 (1%)	7 (1%)

Figure S2. Standardized differences for baseline characteristic comparing LPV/r to other drugs before and after matching

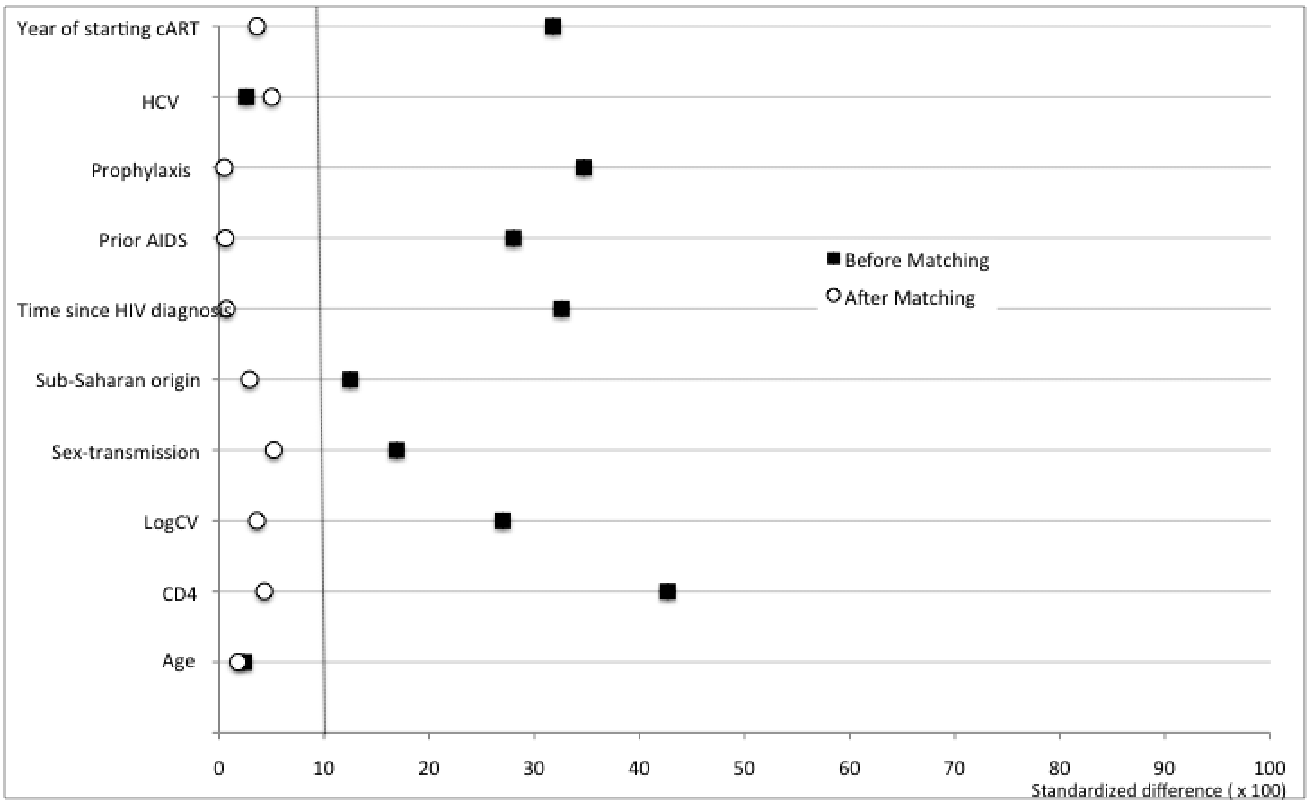

Table S2. Non-AIDS hospitalizations

	LPV/r		EFV, ATV/r		Total	
	N	%	N	%	N	%
Infection non AIDS	64	41.8%	27	32.1%	91	38.4%
Chronic viral hepatitis	9	5.9%	4	4.8%	13	5.5%
Malignancy non AIDS defining	8	5.2%	4	4.8%	12	5.1%
Diabetes Mellitus	2	1.3%	0	0.0%	2	0.8%
Lactic acidosis	0	0.0%	0	0.0%	0	0.0%
MI or other ischemic heart disease	2	1.3%	0	0.0%	2	0.8%
Stroke	1	0.7%	0	0.0%	1	0.4%
Gastro-intestinal hemorrhage	2	1.3%	2	2.4%	4	1.7%
Primary pulmonary hypertension	1	0.7%	0	0.0%	1	0.4%
Chronic obstructive lung disease	1	0.7%	0	0.0%	1	0.4%
Liver failure	2	1.3%	0	0.0%	2	0.8%
Renal failure	1	0.7%	0	0.0%	1	0.4%
Suicide	0	0.0%	0	0.0%	0	0.0%
Substance abuse (active)	2	1.3%	0	0.0%	2	0.8%
Hematological disease	9	5.9%	3	3.6%	12	5.1%
Endocrine disease	1	0.7%	0	0.0%	1	0.4%
Psychiatric disease	5	3.3%	4	4.8%	9	3.8%
Heart or vascular	4	2.6%	2	2.4%	6	2.5%
Respiratory disease	1	0.7%	2	2.4%	3	1.3%
Digestive system disease	5	3.3%	2	2.4%	7	3.0%
Skin and motor system disease	1	0.7%	2	2.4%	3	1.3%
Urogenital disease	2	1.3%	5	6.0%	7	3.0%
Other causes	17	11.1%	16	19.0%	33	13.9%
Unclassifiable causes	3	2.0%	7	8.3%	10	4.2%
Unknown	10	6.5%	4	4.8%	14	5.9%
Total	**153**		**84**		**237**	
